# Open Science, equity and the Brazilian context

**DOI:** 10.1590/S2237-96222022000200001

**Published:** 2022-08-29

**Authors:** Barbara Reis-Santos, Cynthia Braga

**Affiliations:** 1 Rede Brasileira de Pesquisas em Tuberculose, Rio de Janeiro, RJ, Brazil; 2 Instituto Aggeu Magalhães, Fundação Oswaldo Cruz, Recife, PE, Brazil

Open Science, a global movement created by the scientific community, has undertaken efforts aimed at increasing the popularity of scientific knowledge production and making the results of scientific research widely accessible to society. Open Science principles, which aim to make research outcomes freely available through scientific journals or open access repositories, and to promote transparency to their processes, replication and reproducibility, have generally been well received by the scientific community. Its transparent, accessible and collaborative nature is widely recognized, but discussion of potentially reckless aspects for implementation, such as the costs of participation and the need for a favorable policy agenda, are also in focus.^1^

As this "umbrella of strategies" spreads, some questions become more frequent: how can we promote equity from such an inequitable basis, after all? The venue for the "open science party" has been set, but the capacity of the guests to take part in it has not been the same. Surveys show that the article processing charges (APCs), charged by international open access journals have increased sharply and constituted a barrier to the visibility of scientific production of researchers globally.^2^ ^,^ ^3^ Thus, the Brazilian scientific community is among those agents whose capacity to participate is compromised by budget constraints for research and the lack of resources directed to publication fees by funding agencies in Brazil.

Science has been deeply marked by advances and setbacks, in Brazil. Even in the early 2000s, the structural working conditions, regional imbalances, funding options and conflicts between the public and private sectors were already seen as challenges.^4^ The situation has not changed much and, despite an upward trend in federal government spending on science and technology, from 2003 to 2015, this trend was reversed as of 2016, reaching levels below the investments in 2009 by 2020.^5^ This funding setback has imposed heavy burdens on the entire scientific community, reflected in difficulties to maintain institutions and projects, and consequent expansion of barriers, making Brazilian researchers even more distant from the effective implementation of Open Science.

Regarding scientific journals published in Brazil, the scenery is not different. If, on the one hand, Brazil has a significant number of open access journals, thanks to the leadership of the SciELO Program, on the other hand, lack of financial support has caused some journals to start charging APC in recent years and has even put the sustainability of SciELO at risk.^6^ This scenery has been aggravated by the evaluation policy of national postgraduate programs, which favors publication in high impact factor international journals to the detriment of national journals.

In this context of financial constraints and discouragement to both journals published in Brazil and researchers, we have been faced with a health crisis caused by the COVID-19 pandemic. The response of the scientific community was immediate and consistent, gaining prominence even on the international scene. With regard to health care professionals who are responsible for the care of people, the demand for timely access to reliable sources of information became even more evident during the pandemic, whether to obtain data about the risks inherent to work, or for training related to the care provided.

Epidemiology and Health Services journal (*Epidemiologia e Serviços de Saúde* - RESS), given its mission to contribute to the improvement of services offered by the Brazilian National Health System (SUS), considers these professionals as an important part of its target audience. In order to reinforce this approach, a study contribution box was included in the articles published as of 2022, in which the "implications for services", as well as new dissemination strategies are shown. In addition, RESS contributes to the promotion of Open Science, allowing publications free of charge to researchers whose results of analyses and studies have been developed within the SUS. Several other Public Health journals have also invested in science communication strategies, in order to be recognized as a reliable source of information, a movement of the utmost importance, since open access content is promoted, and its evidence is mostly generated in the national context and without language barriers, which often reduces professionals’ access to articles published internationally.

As such, we believe that we are consolidating a path to overcome "publication circuits", in which the scientific community discusses among themselves, however, health professionals are not included. Broadening the discussion on the role of national scientific journals in order to promote the dissemination of knowledge and strengthening of Open Science in the American continent context, or globally, is still necessary, and can contribute to the inclusion of society, in the defense of the scientific community and policies aimed at promoting equity, as a pillar for the implementation of Open Science. We can work in such a way that repercussions of this movement also reach the base of the pyramid, and not only the top, thus, this will result in a condition of more equitable participation in this "party", which is a RESS commitment.

## References

[1] Ross-Hellauer T, Reichmann S, Cole NL, Fessl A, Klebel T, Pontika N (2022). Dynamics of cumulative advantage and threats to equity in open
science: a scoping review. R Soc Open Sci.

[2] Zhang L, Wei Y, Huang Y, Sivertsen G (2022). Should open access lead to closed research ? The trends towards
paying to perform research. Scientometrics.

[3] Kwon D (2022). Open-access publishing fees deter researchers in the global
south. Nature.

[4] Chaimovich H (2000). Brasil, ciência, tecnologia: alguns dilemas e
desafios. Estudos avançados.

[5] De Negri F (2021). Políticas públicas para ciência e tecnologia no Brasil: cenário e
evolução recente.

[6] Garcia LP, Boing AF (2021). Desafios para a sustentabilidade dos periódicos científicos
brasileiros e do Programa SciELO. Cien Saude Colet.

